# Identification of Gedunin from a Phytochemical Depository as a Novel Multidrug Resistance-Bypassing Tubulin Inhibitor of Cancer Cells

**DOI:** 10.3390/molecules27185858

**Published:** 2022-09-09

**Authors:** Sami A. Khalid, Mona Dawood, Joelle C. Boulos, Monica Wasfi, Assia Drif, Faranak Bahramimehr, Nasim Shahhamzehei, Letian Shan, Thomas Efferth

**Affiliations:** 1 Faculty of Pharmacy, University of Science & Technology, Omdurman, Sudan; 2 Department of Pharmaceutical Biology, Institute of Pharmaceutical and Biomedical Sciences, Johannes Gutenberg University, Staudinger Weg 5, 55128 Mainz, Germany; 3 Department of Molecular Biology, Faculty of Medical Laboratory Sciences, Al-Neelain University, 12702 Khartoum, Sudan; 4 The First Affiliated Hospital, Zhejiang Chinese Medical University, Hangzhou 310053, China

**Keywords:** cancer, microtubuli, natural product, network pharmacology, targeted chemotherapy

## Abstract

The chemotherapy of tumors is frequently limited by the development of resistance and severe side effects. Phytochemicals may offer promising candidates to meet the urgent requirement for new anticancer drugs. We screened 69 phytochemicals, and focused on gedunin to analyze its molecular modes of action. Pearson test-base correlation analyses of the log_10_IC_50_ values of 55 tumor cell lines of the National Cancer Institute (NCI), USA, for gedunin with those of 91 standard anticancer agents revealed statistically significant relationships to all 10 tested microtubule inhibitors. Thus, we hypothesized that gedunin may be a novel microtubule inhibitor. Confocal microscopy, cell cycle measurements, and molecular docking in silico substantiated our assumption. Agglomerative cluster analyses and the heat map generation of proteomic data revealed a subset of 40 out of 3171 proteins, the expression of which significantly correlated with sensitivity or resistance for the NCI cell line panel to gedunin. This indicates the complexity of gedunin’s activity against cancer cells, underscoring the value of network pharmacological techniques for the investigation of the molecular modes of drug action. Finally, we correlated the transcriptome-wide mRNA expression of known drug resistance mechanism (ABC transporter, oncogenes, tumor suppressors) log_10_IC_50_ values for gedunin. We did not find significant correlations, indicating that gedunin’s anticancer activity might not be hampered by classical drug resistance mechanisms. In conclusion, gedunin is a novel microtubule-inhibiting drug candidate which is not involved in multidrug resistance mechanisms such as other clinically established mitotic spindle poisons.

## 1. Introduction

Medicinal herbs have been used by mankind for thousands of years. Only with the groundbreaking successes of chemistry since the 19th century have herbs increasingly faded into the background. A milestone in this development was the first isolation of morphine from *Papaver somniferum* by Sertürner in the year 1804 [1]. Today, medicinal herbs still play an important role despite the dominance of chemically synthesized drugs. Repeated analyses of the National Cancer Institute in the USA showed that more than two-thirds of all drugs approved on the market have a reference to natural sources—either those natural substances are directly medicinal active ingredients, chemical derivatives of natural substances are used, or biological principles of action were copied from nature for drug development [2]. In addition to natural product-driven drug development in Western industrialized nations, even today natural products remain essential in many developing countries. According to the World Health Organization (WHO), basic medical care there is based up to 80% on traditional medicine and medicinal herbs [3]. It is estimated that there are about 30,000 medicinal plants out of an entirely estimated number of 270,000 terrestrial plants on earth [4,5]. For this reason, systematic research on medicinal plants and their constituents is still of great importance in pharmacology [6]. Chemotherapy with clinically established anticancer drugs often fails due to the emergence of resistance, of which the multidrug resistance (MDR) phenotype is particularly feared because it results in the simultaneous ineffectiveness of many drugs, thereby drastically reducing the chances of therapeutic success. MDR is characterized by broad cross-resistance to a majority of clinically established anticancer drugs, including anthracyclines, antibiotic anticancer drugs, Vinca alkaloids, taxanes, epipodophyllotoxins, tyrosine kinase inhibitors, and others. As such, the options to treat tumors that are resistant to one drug using another drug are severely limited, with fatal consequences for the patients. MDR can appear as a primary (or inherent) resistance phenomenon (where a tumor is resistant from the beginning on to chemotherapy) and as secondary (or acquired) resistance (where resistance transiently develops during several treatment cycles, leading to full-blown refractoriness at the end of chemotherapeutical treatment). MDR is caused by a drug efflux pump, P-glycoprotein, that belongs to the ATP-binding cassette (ABC) transporter family. P-glycoprotein transports a large array of xenobiotic compounds, including anticancer drugs [7,8]. The clinical failure of chemotherapy due to multidrug resistance stimulated the quest for new cytotoxic compounds (including phytochemicals) that are not substrates of P-glycoprotein, and which thereby have the ability to bypass multidrug resistance and kill multidrug-resistant tumor cells with a similar efficacy to sensitive ones.

In the present study, we screened a total of 69 phytochemicals for their anti-crescent activity against MDR tumor cells. An interesting candidate identified in this analysis is the pentacyclic triterpenoid gedunin. Subsequently, we investigated the molecular mechanisms of action using proteomic and molecular pharmacological methods. We found not only that gedunin killed MDR cells with similar efficiency to drug-sensitive cells, but also that gedunin inhibited microtubules, representing a novel mitotic spindle toxin. Because the efficacy of clinically established microtubule inhibitors such as *Vinca* alkaloids and taxanes is compromised in MDR cells by P-glycoprotein-mediated efflux, gedunin represents an interesting new alternative.

## 2. Results

### 2.1. Activity Screening of 69 Phytochemicals in Leukemia Cells

A total of 69 chemical constituents from medicinal plants were selected at the Faculty of Pharmacy, University of Science & Technology, Omdurman, Sudan. Most of these phytochemicals occur not only in their known stem plants but also in several other plants (Table 1). The characteristics of these phytochemicals as shown in Supplementary Table S1.

**Figure 1:**
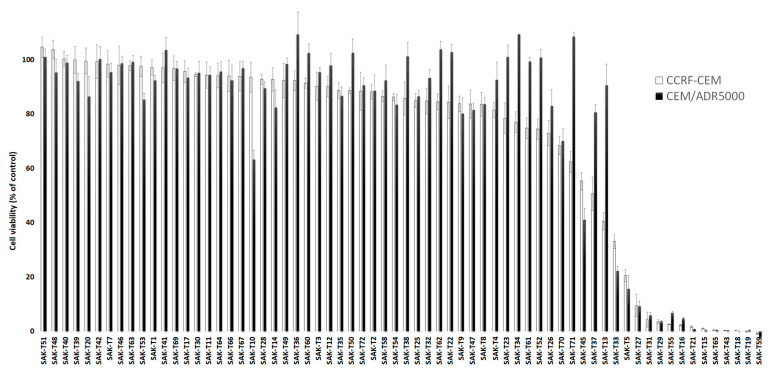
Screening of 69 phytochemicals in drug-sensitive parental CCRF-CEM and multidrug-resistant CEM/ADR5000 cells using the resazurin assay. A fixed concentration of 10 µM of each concentration was used. Shown here are the mean values ± SD of three independent experiments.

These 69 phytochemicals were investigated for their growth-inhibitory potential towards human CCRF-CEM leukemia cells and their multidrug-resistant P-glycoprotein-overexpressing sub-line, CEM/ADR5000, using the resazurin assay. All of the compounds were tested at a fixed concentration of 10 µM. This was a preliminary experiment to separate substances that affect these two cell lines from those that were inactive. As shown in Figure 1, 14 compounds reduced cell viability by more than 60% of the untreated controls. Hence, 20% (14/69) of all of the tested compounds showed inhibitory potential to these cell lines.

**Figure 2:**
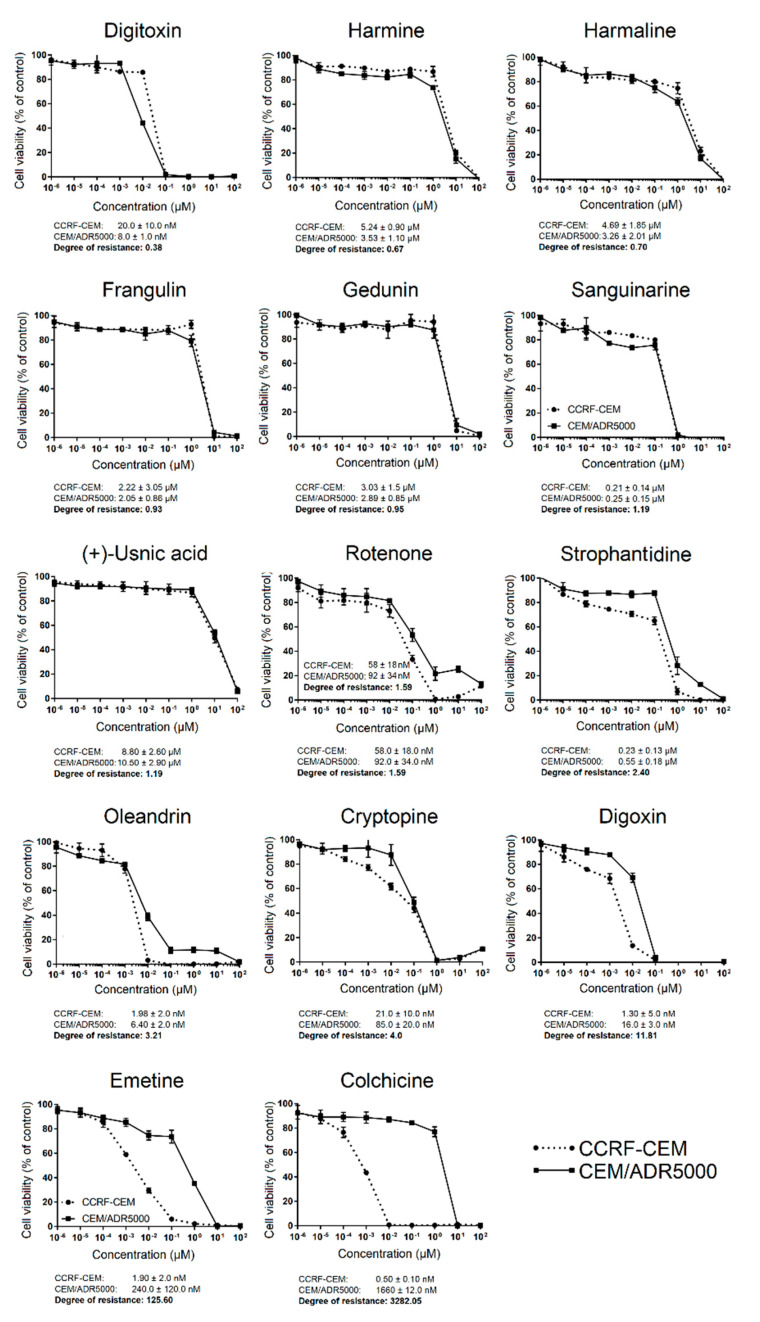
Dose-response curves of 14 selected phytochemicals in drug-sensitive parental CCRF-CEM and multidrug-resistant CEM/ADR5000 cells using the resazurin assay. The IC_50_ values were determined from the dose-response curves, and the degrees of resistance were obtained by dividing the IC_50_ of CEM/ADR5000 cells by the corresponding IC_50_ value of CCRF-CEM cells. Shown here are the mean values ± SD of three independent experiments.

The 14 active compounds were then subjected to a second series of experiments, in which we tested a concentration range from 10^−4^ to 10^2^ µM using the resazurin assay. The dose-response curves of the CCRF-CEM and CEM/ADR5000 cells depicted in Figure 2 were used to calculate the 50% inhibition concentrations (IC_50_), and the IC_50_ values of multidrug-resistant CEM/ADR5000 cells were divided by the IC_50_ values of sensitive parental CCRF-CEM cells to obtain the degrees of resistance to the corresponding phytochemicals. The IC_50_ values of CCRF-CEM cells to the 14 compounds were in a range between 0.005 (±0.001) to 8.80 (±2.60) µM, and those of CEM/ADR5000 were in a range between 0.0064 (±0.002) to 10.5 (±2.90) µM. CEM/ADR5000 was sensitive to seven compounds with degrees of resistance below 1.2 (digitoxin, harmine, harmaline, frangulin, gedunin, sanguinarine, and (+)-usnic acid). The weak cross-resistance of CEM/ADR5000 cells with degrees of resistance between 1.2 and 10 was observed to four compounds (rotenone, strophantidine, oleandrin, and cryptopine). Furthermore, CEM/ADR5000 cells were highly cross-resistant to three compounds (digoxin, emetine, and colchicine), with degrees of resistance above 10.

Gedunin was selected as an example to test the cytotoxicity against non-malignant cells. Normal leukocytes (peripheral blood mononuclear cells) isolated from buffy coat were tested in the same dose range as CCRF-CEM and CEM/ADR cells, and no inhibition was measured (Supplementary Figure S1).

### 2.2. Molecular Docking of Selected Phytochemicals to P-Glycoprotein

**Figure 3:**
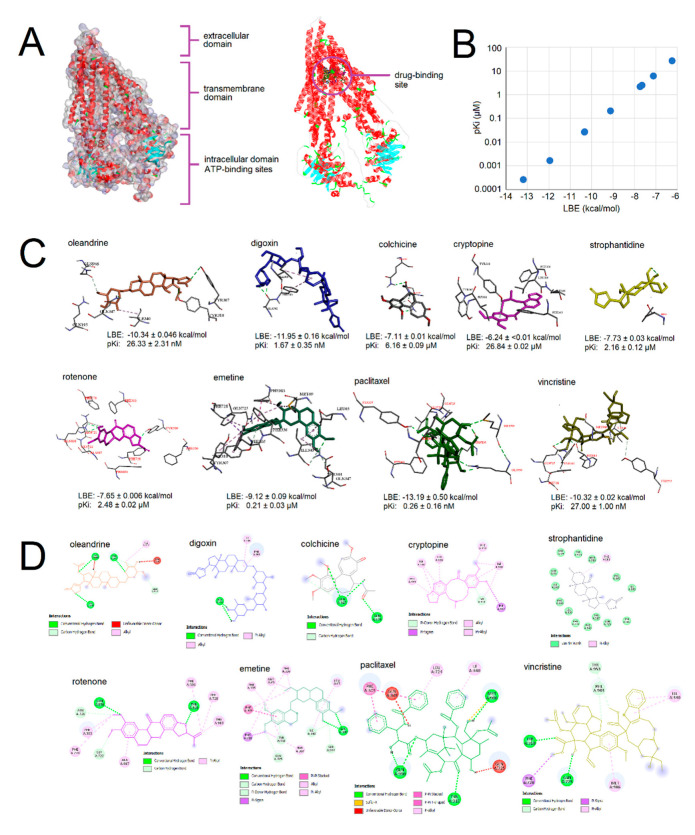
Molecular docking of seven selected phytochemicals to a human homology model of P-glycoprotein in the open conformation. (**A**) Extracellular domain, transmembrane domain, intracellular domain with ATP-binding sites, and drug-binding site of P-glycoprotein. (**B**) Correlation of lowest binding energies (LBE, kcal/mol) and predicted inhibition constants (pKi, µM) of seven selected phytochemicals. (**C**) Binding orientation of the selected phytochemicals at the drug-binding site. (**D**) Interaction of the selected phytochemicals with amino acid residues at the drug binding site. The established anticancer drugs paclitaxel and vincristine were chosen as control drugs, as they are well-known to be transported by P-glycoprotein.

In order to investigate whether the cross-resistance of CEM/ADR5000 observed to some of the 14 phytochemicals may be due to P-glycoprotein, we performed molecular docking analyses. Figure 3A shows the different domains of P-glycoproteins and also the drug-binding site. In Figure 3B, we correlated the lowest binding energies (LBE, kcal/mol) and predicted the inhibition constants (pKi, µM) of seven selected compounds (oleandrine, digitoxin, colchicine, cryptopine, strophantidine, rotenone, and emetine). The lowest binding energy values ranged from −11.95 kcal/mol (digoxin) to −6.24 kcal/mol (cryptopine), and the predicted inhibition constants ranged from 1.67 nM (digoxin) to 26.84 µM (cryptopine). Paclitaxel and vincristine were added as positive controls, as they are well-known P-glycoprotein substrates. We obtained a linear regression line with a strong correlation of both parameters (R^2^ = 1.0). This correlation analysis was performed as quality control for our in silico calculations. Figure 3C shows that the selected compounds were all bound to the drug binding site of P-glycoprotein but at different orientations.

### 2.3. Activity Profiling of Selected Phytochemicals in Cell Lines of Diverse Tumor Types

**Figure 4:**
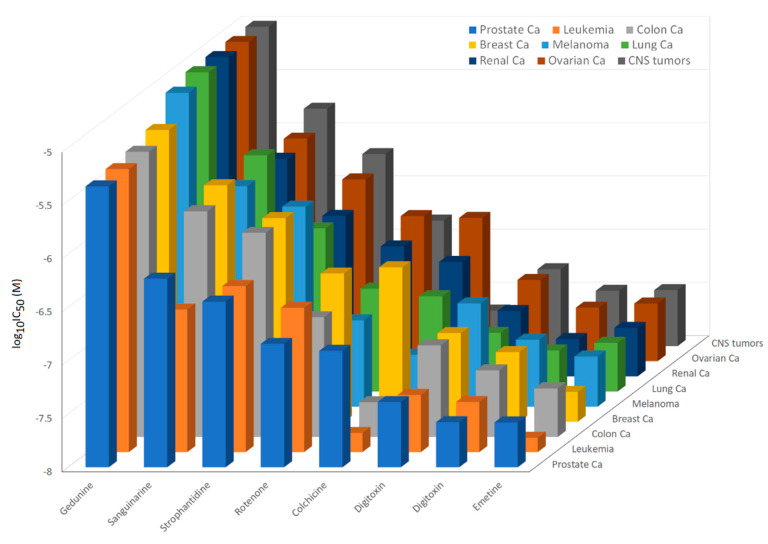
Cytotoxicity of eight selected phytochemicals to the NCI panel of the NCI consisting of cell lines derived from different tumor origins (leukemia; melanoma; brain tumors; carcinoma of the prostate, colon, breast, lung, kidney, or ovary). Shown are the mean log_10_IC_50_ values for each tumor type and each compound.

So far, the experiments focused on sensitive and multidrug-resistant leukemia cells, and we did not know about the activity of the 14 phytochemicals on tumor cell lines from other tumor types. For this reason, we inspected the database of the Developmental Therapeutics Program of the National Cancer Institute (Bethesda, MA, USA; https://dtp.cancer.gov, accessed on 9 September 2022) regarding whether these compounds are deposited there. Nine of the 14 compounds were deposited in this database. These compounds were investigated for their cytotoxic activity by means of a sulforhodamine B assay towards cell lines of different tumor types (leukemia; melanoma; brain tumors; carcinoma of the colon, ovary, breast, kidney, lung, or prostate), in order to determine which tumor types are most sensitive or most resistant to which of the eight phytochemicals. The responsiveness of the cell lines from different tumor types was plotted as mean log_10_IC_50_ values in Figure 4. The cell lines of all of the tumor types were most sensitive to emetine, followed by digoxin and digitoxin. The fact that not that many differences in the mean log_10_IC_50_ values were observed may speak for the hypothesis that these compounds are highly toxic and that they kill cancer cells in a rather non-specific manner. The mean log_10_IC_50_ values were all lower than −7 M. On the other hand, strophantidine and gedunin showed higher mean log_10_IC_50_ values (between −6.5 to −5 M), but it was possible to better differentiate between the tumor types. Gedunin was more sensitive to pancreas carcinoma and leukemia cell lines, but more resistant to brain tumor or ovarian carcinoma cell lines. Hence, we hypothesized that the activity of gedunin might be more specific than those of emetine and digitoxin.

### 2.4. Acute and Chronic Toxicity of Selected Phytochemicals

In order to prove this hypothesis, we mined the PubChem database for the toxicities of the eight selected phytochemicals (Table 2). Most compounds show acute toxicities, such as cardio, hepato- nephron-, and/or gastrointestinal toxicity. Some compounds also provoke pulmonary, neuro-, and/or behavioral toxicity. These severe and partwise life-threatening toxicities are not observed with gedunin, although gedunin is also not free from acute toxic reactions, and dermal toxicity has been reported. Concerning chronic toxicity, some of the other compounds can be mutagenic, carcinogenic, teratogenic, or irreversibly cardiotoxic. No chronic toxicity is recorded for gedunin. Taken together, it can be concluded that gedunin is safer than the other phytochemicals analyzed.

**Figure 5:**
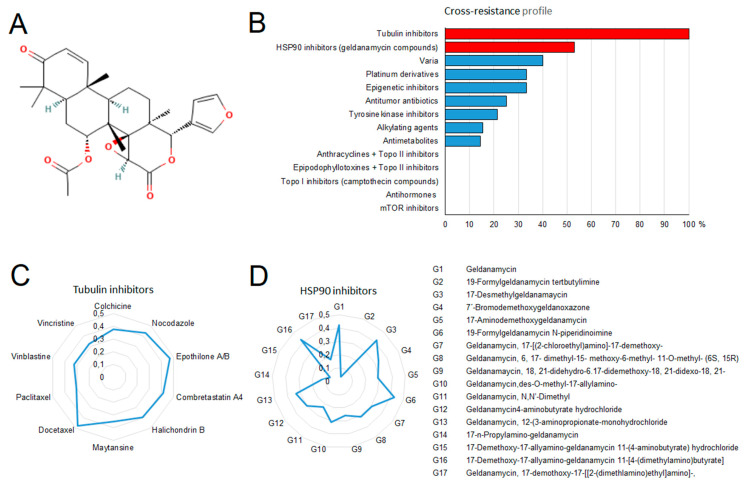
Cross-resistance profiling of gedunin to standard anticancer drugs. (**A**) Chemical structure of gedunin. (**B**) Percentage of standard anticancer drugs from different pharmacological classes that significantly correlated to the responsiveness of NCI tumor cell lines to gedunin (with *p* < 0.05 and *r* > 0.3 as cutoff values). Oncobiograms of (**C**) tubulin-inhibiting drugs or (**D**) geldanamycin derivatives correlating to the response of NCI tumor cell lines to gedunin.

As a next step, we correlated the log_10_IC_50_ values of gedunin for 55 tumor cell lines to those of 91 standard anticancer agents from different pharmacological classes. As shown in Figure 5B, the log_10_IC_50_ values of gedunin correlated to all of the tubulin-inhibiting compounds. Previously, gedunin was described to inhibit the heat shock protein 90 (HSP90) by binding to the co-chaperone p23 [9]. As a positive control, we, therefore, correlated the log_10_IC_50_ values for gedunin, and also for those of the well-known HSP90 inhibitor geldanamycin and 17 of its derivatives. The cellular responsiveness of gedunin correlated with nine out of 17 (=53%) of the geldanamycin derivatives in the NCI cell line panel (*p* < 0.05). Fewer correlations were found to compounds from other drug classes (platinum compounds, epigenetic inhibitors, antitumor antibiotics, tyrosine kinase inhibitors, alkylating agents, and antimetabolites). No correlations were found to anthracyclines, epipodophyllotoxins, or other DNA topoisomerase II inhibitors, or to DNA topoisomerase I inhibitors (camptothecin compounds), antihormones, or mTOR inhibitors. A detailed analysis of the correlation of gedunin with tubulin inhibitors and HSP90 inhibitors (geldanamycin derivatives) is shown in Figure 5C,D.

### 2.5. Effect of Gedunin on the Microtubule Network

**Figure 6:**
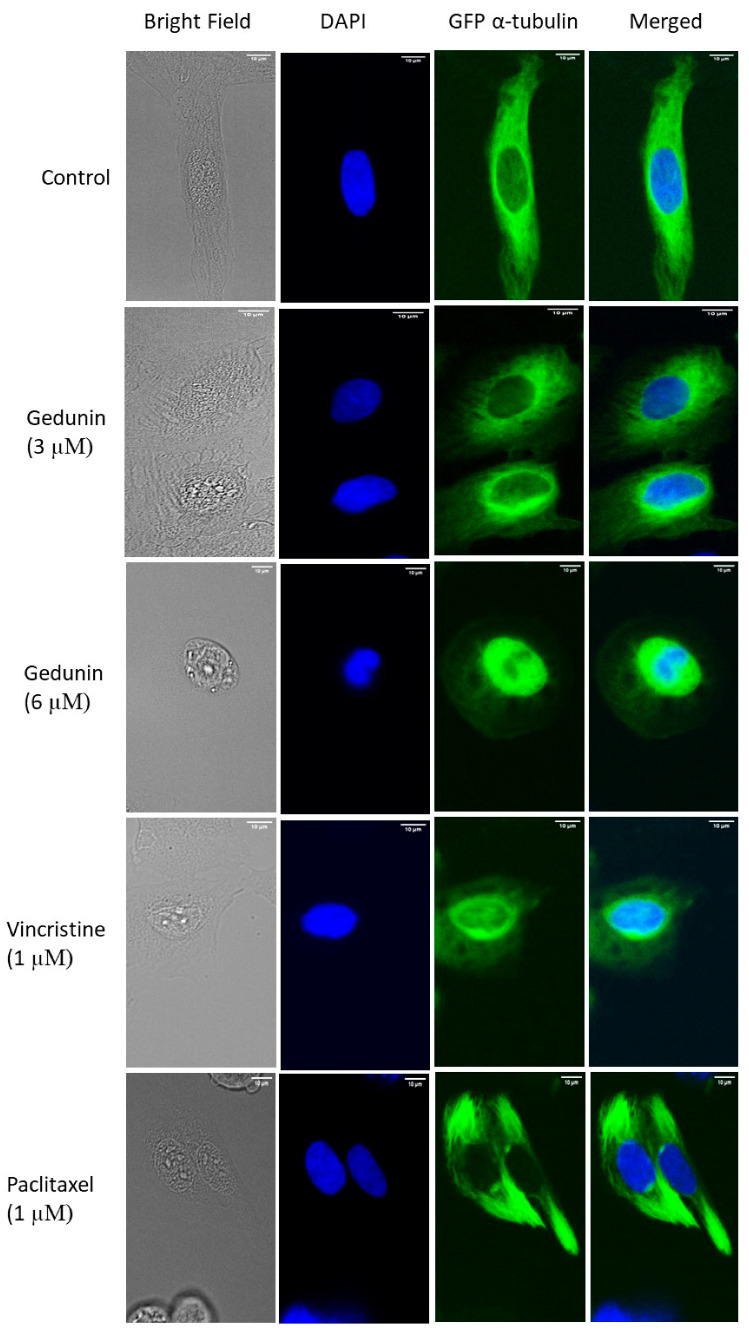
Disruption of the microtubule network in gedunin-treated U2OS cells. Micrographs of fixed U2OS cells were taken after 24 h of treatment with DMSO, various gedunin concentrations, vincristine, and paclitaxel. The microtubules were imaged at 40× magnification using an AF7000 widefield fluorescence microscope (scale bars = 10 µm). The images were merged with DAPI (blue) to represent the nucleus.

In order to investigate the effect of gedunin on microtubules, U2OS human osteosarcoma cells transfected with α-tubulin-GFP fusion protein were subjected to different gedunin concentrations (3 µM or 6 µM) for 24 h. As shown in Figure 6, the tubulin network in control cells was well polymerized, and it was obviously perceived by the considerable dispersion of tubulin across the cytoplasm, generating a solid intracellular network. On the contrary, a disintegrated tubulin network was noticed in gedunin-treated cells, and this effect was similar in vincristine-treated cells. Unlike paclitaxel-treated cells, in which tubulin appeared stiff, gedunin and vincristine decreased the expansion of microtubules at the periphery and increased the mass of tubulin around the nucleus. Furthermore, the thickness of the microtubules at the extremities of gedunin-treated cells was reduced compared to negative control cells. Altogether, these data indicated that, like vincristine, gedunin restrained the polymerization of the microtubule network.

### 2.6. Molecular Docking of Gedunin to β-Tubulin

**Figure 7:**
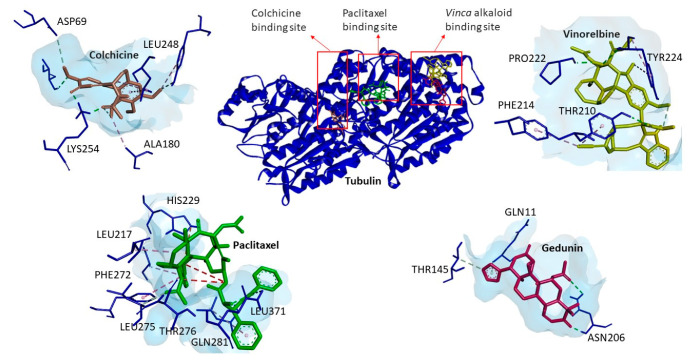
Molecular docking of gedunin to human α- and β-microtubules using AutoDock4.2.6. Gedunin was preferentially bound to the *Vinca* alkaloid binding site but not to the taxane or colchicine binding sites.

Defined molecular docking was carried out in order to study the binding affinity of gedunin to the best-known microtubule binding sites, i.e., the *Vinca* alkaloid binding site, the colchicine binding site, and the taxane binding site. As shown in Table 3, gedunin exhibited a stronger binding affinity to the *Vinca* alkaloid binding site compared to the colchicine and taxane binding sites. Using Discovery Studio Visualizer, we showed that gedunin interacted in the same binding site of the *Vinca* alkaloid as vinorelbine, although at different amino acid residues (Figure 7). This indicates that gedunin might inhibit microtubule polymerization.

### 2.7. Proteome Profiling of Gedunin

The expression of 3171 proteins, as measured by mass spectrometry and deposited at the database of the Developmental Therapeutics Program of NCI, USA, were correlated to the log_10_IC_50_ values of gedunin for 55 tumor cell lines using COMPARE analysis. Based on Pearson’s correlation test, a list of 40 proteins was assembled out of a total 3171 proteins consisting of the top 20 positively and the top 20 inversely correlating proteins with the log_10_IC_50_ values for gedunin. These 40 proteins and their biological functions are compiled in Supplementary Table S2.

**Figure 8:**
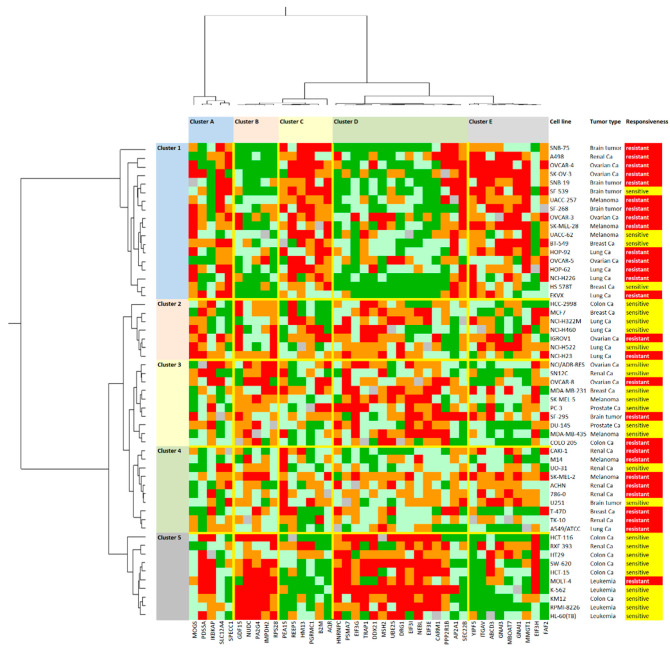
Heat map and agglomrative cluster analyses of the protein expression in the response of 55 tumor cell lines to gedunin (log_10_IC_50_). The proteins are labeled as numbers at the top of the heatmap. The proteins assigned to each number are given in Supplementary Table S2. The cell lines, their tumor origins, and their sensitivity/resistance to gedunin are shown at the right side of the heat map. Cell lines with individual log_10_IC_50_ values smaller than the median value of all of the 55 cell lines tested (−5.125 M) were defined as being sensitive, while all of the others with log_10_IC_50_ values above the median were defined as being resistant to gedunin. The cluster analysis (Ward method) separated the proteins into five clusters (clusters A-E) and the cell lines also into five clusters (clusters 1–5). The cell lines were clustered according to their degrees of relatedness to each other on the basis of their protein expression included in the analysis. Color code: red, 0–25% quartile; orange, 26–50% quartile; grey, median value; light green, 50–75% quartile; dark green, 76–100% quartile.

Afterward, we performed an agglomerative cluster analysis according to the Ward method with the cellular expression of these 40 proteins in the first dimension and the log_10_IC_50_ values for gedunin in the second dimension. This two-dimensional clustering approach resulted in a color-coded heat map (Figure 8). Five major clusters were obtained for the 40 proteins (clusters A–E), and another five clusters appeared for the 55 tumor cell lines tested (clusters 1–5). The cellular responsiveness of the cell lines to gedunin was categorized by defining the cell lines as being gedunin-sensitive if their individual log_10_IC_50_ value was smaller than the median value across all of the 55 cell lines, and as being gedunin-resistant if the individual log_10_IC_50_ value was higher than the median. Then, we calculated whether the distribution of sensitive and resistant cell lines was statistically different between the cell line clusters by using the χ^2^ test. Indeed, we found that clusters 2, 3, and 5 mainly contained gedunin-sensitive cell lines, whereas clusters 1 and 4 mainly consisted of gedunin-resistant cell lines (*p* = 0.002; Table 4, upper panel). This indicates that the expression profile of this set of 40 proteins indeed enabled us to predict gedunin’s sensitivity and resistance in this panel of cell lines.

Next, we looked in greater detail at the tumor types which tended to be more susceptible to gedunin than others (Table 4, lower panel). The majority of cell lines derived from leukemia and carcinoma of the breast, prostate, or colon tended to be gedunin-sensitive, whereas cell lines from brain tumors, melanoma, and carcinoma of the kidney, ovary, or lung were rather gedunin-resistant. This relationship only showed a trend, but did not reach statistical significance (*p* = 0.076).

### 2.8. Proteomic Canonical Pathway and Network Analyses of Gedunin

**Figure 9:**
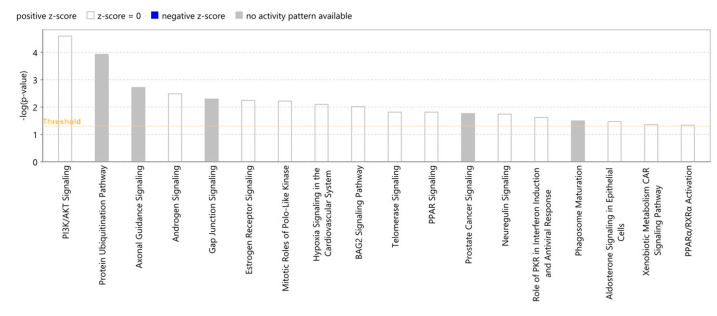
Canonical signaling pathways were predicted by using the proteomic data of 40 proteins in a panel of 55 NCI tumor cell lines and IPA™.

**Figure 10:**
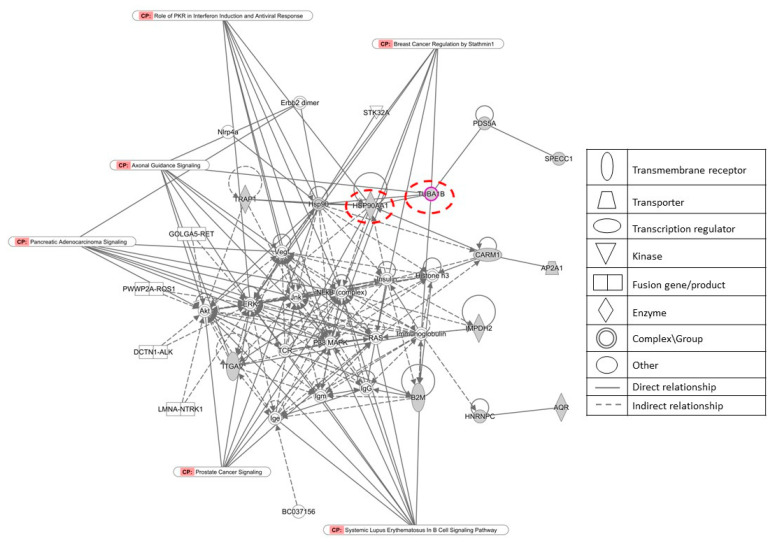
Network analysis using the proteomic data of 40 proteins in a panel of 55 NCT tumor lines and IPA™.

The proteomics data compiled in Supplementary Table S2 were subjected to Ingenuity Pathway Analysis (IPA™). We first investigated functional canonical signaling pathways to see whether gedunin affects known signaling pathways. We found that PI3K/AKT signaling, the protein ubiquitinated pathway, axonal guidance signaling, androgen signaling, and gap junction signaling, among others, were affected by gedunin (Figure 9). Because several pathways were involved, we speculated that an individual interaction network of proteins—rather than single, defined pathways—may play a role. Therefore, we performed network analyses using IPA™. Interestingly, the network interactome in Figure 10 shows not only the subset of 40 proteins but also that the two gedunin targets (HSP90 and β-tubulin) were connected in this gedunin-specific interaction network.

### 2.9. Drug Resistance Profiling of Gedunin

Finally, we were interested in whether gedunin is involved in the drug resistance phenotypes not only of P-glycoprotein but also of other ABC-transporters (ABCB5, ABCC1, and ABCG2), as well as drug resistance mechanisms apart from ABC-transporters, such as the oncogene EGFR, the tumor suppressors TP53 and WT1, the heat shock protein HSP90, glutathione S-transferase π, and the proliferation rate of the cell lines (Table 5). We did not observe statistically significant correlations of the log_10_IC_50_ values for gedunin and any of the resistance parameters except for EGFR expression. This indicates that the overexpression of this oncogene was associated with resistance to gedunin, while this phytochemical is not involved in all of the other drug resistance mechanisms.

## 3. Discussion

We investigated 69 phytochemicals derived from medicinal plants. Using parental, drug-sensitive CCRF-CEM, and multidrug-resistant CEM/ADR5000 cells, we found that the viability of 14/69 (=20%) of the compounds was inhibited. A hit rate of up 20% is reasonable and compares to similar studies with medicinal plant extracts and isolated natural products in the past from us and others [10,11,12]. We focused on eight of these 14 compounds and found strong toxicities for most of them, largely disqualifying them for further consideration in anticancer drug development. This was not the case with gedunin; we therefore investigated this compound in more detail. Gedunin is a triterpene found in diverse plants, including *Azadirachta indica*, several species of the Meliaceae family, and in other plants (Table 1). Gedunin possesses a wide variety of bioactivities, including insecticidal, antiplasmodial, antibacterial, antiallergic, anti-inflammatory, anticancer, and neuroprotective effects. It was initially described as an inhibitor of heat shock protein 90, but the great diversity of different bioactivities makes further mechanisms of action likely [13].

Exploiting the database of the Developmental Therapeutics Program of the NCI, USA, revealed that gedunin may act like a tubulin inhibitor. This was a surprising finding, as gedunin was described in the literature as an HSP90 inhibitor [14]. It is quite common that natural products are multi-specific and have several targets [15]. Therefore, we investigated this hypothesis in more detail. Correlation analyses showed that the percentage of tubulin inhibitors correlating with the cellular responsiveness of gedunin was much higher than those of HSP90 inhibitors. The generation of testable hypotheses on the mechanisms of action of investigational drugs by correlating their IC_50_ values with those of established anticancer drugs with well-known modes of action was developed by the NCI (Bethesda, MA, USA) [16,17]. Therefore, we utilized this strategy for our investigation on gedunin.

Strong evidence came from our studies with confocal microscopy. Gedunin showed a similar pattern of destruction of the microtubule network as vincristine. Because vincristine is known to inhibit tubulin polymerization and paclitaxel is known to inhibit tubulin depolymerization, we concluded that gedunin inhibits tubulin polymerization, rather than depolymerization. Furthermore, we observed that gedunin was bound with high affinity to β-tubulin to the *Vinca* binding site but not the taxane binding site in molecular docking studies. This result fits nicely with the data obtained by confocal microscopy demonstrating that gedunin distorted the microtubule network similarly to vincristine but not to paclitaxel. Another result speaking of the interaction of gedunin with microtubules was its arrest of tumor cells in the G2/M phase of the cell cycle [18]. Because microtubules are crucial structures to form the mitotic spindle, microtubule inhibitors such as *Vinca* alkaloids, taxanes, and others usually induce G2/M arrest in cancer cells. Therefore, we conclude from all of these different lines of evidence that gedunin represents a tubulin inhibitor, and that we have identified β-tubulin as a novel target of gedunin in addition to its well-known target, co-chaperon p23/HSP90.

It is common sense that the drug responses of tumor cells is determined by gene expression, e.g., [19,20,21]. The assembly of microarray-based gene expression in cluster image maps (CIMs, or so-called ‘heat maps’) allows us to differentiate sensitive from resistant tumors on the basis of their gene expression. This concept was pioneered by a consortium of the Harvard Medical School, NCI (USA), and other research institutes [22,23]. In past years, we applied this strategy to natural products, e.g., [24,25,26]. While there is a plethora of data using this approach for transcriptomic mRNA expression, there are fewer data generated for proteomic data in this context. Here, we took advantage of the proteomic expression analysis of 3171 proteins in the NCI panel of tumor cell lines [27]. Agglomerative cluster analysis-based heat map generation with proteomic data allowed us to predict the sensitivity or resistance of 55 NCI cell lines to gedunin. We used a series of 40 selected proteins with diverse biological functions to generate a proteomic heat map. The activity of a drug is not only determined by its actual targets (in the case of gedunin, β-tubulin and co-chaperon p23/HSP90) but also by other mechanisms upstream or downstream of them [28]. The purpose of network pharmacology is to identify the interaction networks that explain drug response in its full complexity [29]. The gedunin-relevant subset of 40 out of 3171 proteins consisted of cell surface receptors, intracellular signal transducers, apoptosis regulators, proteins involved in metabolic pathways, cell stress-related proteins, and others. In general, proteins interact not only within well-characterized, defined signaling pathways but also in individual, complex networks. Such interactomes may be interesting for the unravelling of the modes of action of compounds such as gedunin. Based on the known protein functions and interactions, novel drug-related networks can be constructed with the help of bioinformatical tools. In the present study, we identified a gedunin-specific network by using IPA™, which included not only the 40 proteins from the proteomic analysis but also the two gedunin targets, HSP90 and β-tubulin. This indicates that gedunin’s activity against cancer cells may be defined by a complex molecular interactome, rather than linear pathways.

The main problem of all of the anticancer drugs is the development of resistance [30,31]. P-glycoprotein is a well-known drug efflux transporter in cancer cells, expelling a large number of anticancer drugs from diverse pharmacological classes including, *Vinca* alkaloids and taxanes. As such, P-glycoprotein is a typical upstream mechanism mediating multidrug resistance. Therefore, novel anticancer drugs have to be developed that are not recognized as substrates by P-glycoprotein. We investigated the 14 cytotoxic compounds out of the total 69 phytochemicals, and found that multidrug-resistant CEM/ADR5000 cells exerted weak or strong cross-resistance to seven of these substances but not to the other seven phytochemicals. P-glycoprotein and other ABC transporters are not only expressed in tumors but also in normal tissues [32,33] in order to detoxify the body from harmful xenobiotics taken up with food. Therefore, it comes as no surprise that we found several phytochemicals that are presumably P-glycoprotein substrates in multidrug-resistant cells, even if these cells were not in contact before. It was pleasing that gedunin was similarly active against both sensitive and multidrug-resistant tumors, indicating that this natural product is not a substrate of P-glycoprotein.

## 4. Materials and Methods

### 4.1. Isolation of the Characterization of Gedunin

The ground bark of *Azadirachta indica* A. Juss. (2 kg) was percolated for 24 h with refluxing 70% MeOH. The MeOH was evaporated in a rotary evaporator and diluted with H_2_O. The removal of lipophilic impurities was accomplished by repeated distribution between 2 L petroleum ether. The remaining aqueous extract was lyophilized. The lyophilizate was mixed with Si gel 60 (63-230 Merck) and chromatographed on a Si gel column. The column was eluted first with a petroleum ether–ethyl acetate solvent system. The fractions were eluted with petroleum ether–ethyl acetate (70:30) followed by a (60:40) solvent system, which was eventually pooled and concentrated to give 4.2 g of the fraction. This fraction was again subjected to small Si gel 60 column chromatography to isolate 2.0 g gedunin in a petroleum ether–ethyl acetate (65:35) solvent system. The purity of gedunin was monitored by TLC (Kieselgel60, F245, Merck) using petroleum ether–ethyl acetate (60:40) as a developing solvent system. The separation was monitored by TLC (Kieselgel60, F245, Merck) with petroleum ether/EtOAc. Gedunin was crystallized from MeOH, mp 220°, [α]^20^D -44 (CHCI_3_). The full chemical characterization by modern spectroscopic techniques is available [34].

### 4.2. Cell Lines

Drug-sensitive parental CCRF-CEM leukemia cells and their P-glycoprotein-overexpressing multidrug-resistant resistant subline CEM/ADR5000 cells were obtained from Prof. Axel Sauerbrey (University of Jena, Jena, Germany). The culture conditions and the multidrug-resistance phenotype of CEM/ADR5000 cells were previously described [35,36,37].

U2OS-GFP-α-tubulin cells were stably transfected with a GFP fusion construct of *α*-tubulin. The cell line was kindly provided by Joachim Hehl, Light Microscopy Centre, ETH Zurich.

A panel of 55 human tumor cell lines was described [38] and used by the Developmental Therapeutics Program of NCI for drug screening purposes. The cell lines were of diverse origin (leukemia, melanoma, brain tumors, and carcinoma of the lung, colon, kidney, ovary, breast, or prostate). The results of the drug screening (log_10_IC_50_ values obtained by a sulforhodamine 123 assay) and transcriptomic and proteomic expression data were deposited at the NCI website (https://dtp.cancer.gov; accessed on 9 September 2022).

### 4.3. Resazurin Assay

The cell viability of tumor cells upon treatment with 69 phytochemicals, including gedunin, was assessed using the resazurin reduction assay. The procedure was previously described [39,40]. In preliminary experiments, a fixed concentration of 10 µM of the test compounds was applied. Compounds were considered as being active when they reduced cell viability by more than 50%. These substances were then selected to generate dose-response curves applying a concentration range from 0.001 to 100 μM. The concentrations inhibiting cell viability by 50% (IC_50_) were calculated using Graph Prism 6 software (La Jolla, CA, USA). Each experiment was performed independently three times, with six parallel measurements each.

### 4.4. Agglomerative Cluster Analyses of Proteomic Expression Data

The Pearson correlation test and agglomerative cluster analyses were performed using the mass-spectrometry-based proteomic expression data of 55 cell lines from NCI (https://dtp.cancer.gov; accessed on 1 September 2022) [27] to generate a rank-ordered list consisting of the top 20 proteins that directly and the top 20 proteins that inversely correlated with the resistance gedunin based on the log_10_IC_50_ values of the cell lines. Heat maps based on agglomerative clustering according to the Ward method were generated using CIM miner software (https://discover.nci.nih.gov/cimminer/oneMatrix.do; accessed on 9 September 2022) based on the total within-cluster sum of squares [41]. Ingenuity Pathway Analysis (IPA™, Qiagen, Hilden, Germany) was used for canonical pathway and network analyses.

### 4.5. Confocal Microscopy of the Microtubule Network

U2OS human osteosarcoma cells transfected with α-tubulin-GFP construct (30,000 cells/well) were seeded in a µ-Slide 8 Well (ibidi, Gräfelfing, Germany). Cells were placed in a 37 °C/5% CO_2_ incubator overnight; then, they were treated with 3 µM (1 × IC_50_) and 6 µM (2 × IC_50_) gedunin. DMSO was used as a negative control, vincristine (1 µM) was used as a positive control that inhibits polymerization, and paclitaxel (1 µM) was used as a positive control that inhibits depolymerization. After 24 h of treatment, the cells were washed with PBS and fixed with 4% paraformaldehyde for 15 min. Afterward, the cells were washed twice with PBS, and their nuclei were stained with 4′,6-diamidino-2-phenylindole DAPI (1 µg/mL) (Sigma-Aldrich, Darmstadt, Germany) for 5 min at room temperature. Subsequently, the cells were washed with PBS twice to get rid of any excessive staining. The cells were then mounted with ibidi mounting medium (ibidi) and visualized with an AF7000 widefield fluorescence microscope (Leica Microsystems, Wetzlar, Germany). GFP and DAPI were excited with the blue laser (470 nm). GFP emitted light at 525 nm emission; however, DAPI emitted light at 447 nm. Finally, the fluorescent images were analyzed using Fiji ImageJ software (National Institutes of Health, Bethesda, MD, USA) [42].

### 4.6. Molecular Docking

Seven selected phytochemicals (colchicine, cryptopine, digoxin, emetine, oleandrin, rotenone, and strophantidine) were docked to a human homology model of the X-ray diffraction structure of murine P-glycoprotein (PDB ID: 4M1M), and gedunin was docked to the cryo-EM structure of human α- and β-microtubules (PDB ID: 5N5N) using AutoDock 4.2.6 to calculate the binding affinity in silico in a defined docking approach. For docking to P-glycoprotein, a grid box was laid over the pharmacophore at the inner-channel side. Vincristine and paclitaxel were taken as positive controls, as they are known P-glycoprotein substrates. For the docking of gedunin to β-tubulin, grid boxes were laid over the three distinct binding sites of β-tubulin for Vinca alkaloids, taxanes, and colchicine. Vinorelbine, paclitaxel, and colchicine were used as control compounds. A Lamarckian algorithm was used for the docking calculations. Docking results including the lowest binding energy (LBS, kcal/mol) and predicted inhibition constants (pKi, µM) were obtained from the docking log (dlg) file. Three independent dockings were performed with 250,000 runs each to obtain the mean values ± SD. Amino acids involved in ligand interactions were generated by AutoDockTools 1.5.7 (ADT). The docking figure visualization was performed by VMD software.

### 4.7. Statistical Analyses

The COMPARE analysis is based on Pearson’s correlation test. The χ^2^ test was applied to test the statistically significant difference of the observed frequencies in cross tabulation with several categories. These statistical calculations were performed using the WinSTAT program (Kalmia Inc., CA, USA). For heat map construction, the agglomerative cluster analysis method of Ward was performed by using CIM miner software (https://discover.nci.nih.gov/cimminer/oneMatrix.do; accessed on 9 September 2022) based on the total within-cluster sum of squares [41].

## 5. Conclusions

In conclusion, we identified gedunin as a natural product that has the same target as clinically well-established tubulin inhibitors. However, in contrast to them, gedunin is not subject to the multidrug resistance phenotype. This may qualify gedunin as a promising candidate for further drug development.

## Figures and Tables

**Table 1:** Trivial names of 69 phytochemicals and the medicinal plants they are derived from.

Code	Compound	Occurrence in Plants
SAK-T-01	(-)-Scopolamine (hydrochloride)	*Brugmansia* spec., *Datura* spec., *Hyoscyamus* spec., *Mandragora officinarum, Duboisia* spec., *Scopolia carniolica, Physochlaina orientalis*
SAK-T-02	(+)-Bicuculline	*Dicentra cucullaria, Adlumia fungosa, Corydalis* spec, *Fumaria* spec., *Dicentra peregrina*
SAK-T-03	(+)-Laudanosine	*Papaver somniferum, Glaucium flavum, Hazomalania voyronii*
SAK-T-04	(+)-Tubocurarine	*Chondrodendron tomentosum*
SAK-T-05	(+)-Usnic acid	*Ramalina fraxinea, Dimelaena oreina, Nephroma laevigatum, Xanthoparmelia tinctina,* diverse species of the genera *Usnea, Cladonia, Hypotrachyna, Lecanora, Ramalina, Evernia, Parmelia,* and *Alectoria*
SAK-T-07	18-β-Glycyrrhetinic acid	*Glycyrrhiza glabra*
SAK-T-08	3,5-Dimethoxy-4-hydroxy cinnamic acid	*Coffea* spec., *Prunus cerasus, Posidonia oceanica, Lithospermum erythrorhizon, Zea mays, Avena sativa, Triticum aestivum*, *Vitis vinifera*
SAK-T-09	Alloptaeroxylin	*Spathelia sorbifolia*
SAK-T-10	Apigenin	*Lawsonia inermis, Matricaria chamomilla, Verbascum lycnitis, Carex fraseriana, Vernonanthura nudiflora, Salvia* spec., *Dahlia* spec., *Apium* spec.
SAK-T-11	Aristolochic acid	*Aristolochia* spec., *Asarum* spec., *Thottea duchartrei*
SAK-T-12	Barbaloin	*Aloe* spec., *Rhamnus purshiana*
SAK-T-13	Berberine	*Berberis vulgaris, Coptis chinensis, Hydrastis canadensis*
SAK-T-14	Boldine	*Peumus boldis, Lindera* spec., *Licaria triandra, Neolitsea acuminatissima, Damburneya salicifolia, Actinodaphne acuminata*
SAK-T-15	Colchicine	*Colchicum autumnale*
SAK-T-16	Cryptopine	*Argemone mexicana, Fumara* spec., *Papaver* spec., *Arctomecon humilis*
SAK-T-17	Cumarin	*Melilotus officinalis, Galium odoratum, Prunus mahaleb, Dipteryx odorata, Cinnamomum* spec, *Phoenix* spec., and others
SAK-T-18	Digitoxin	*Digitalis purpurea*
SAK-T-19	Digoxin	*Digitalis lanata*
SAK-T-20	Diosgenin	*Dioscorea* spec., *Ophiopogon intermedius, Dracaena draco, Trigonella foenum graecum, Solanum* spec., *Asparagus officinalis, Allium cepa, Hellenia lacera*
SAK-T-21	Emetine (dihydrochloride)	*Carapichea ipecacuanha, Hedera helix, Alangium longiflorum*
SAK-T-22	Emodin	*Polygonum cuspidatum, Polygonum multiflorum, Rheum* spec., *Rhamnus* spec.
SAK-T-23	Emodin anthrone	*Rhamnus prinoides, Paeonia emodi, Rumex acetosa*
SAK-T-25	Esculetin	*Fraxinus* spec., *Artemisia eriopoda, Euphorbia decipiens, Phellodndron amurense, Salvia euphratica*
SAK-T-26	Esculin	*Fraxinus* spec., *Artemisia eriopoda, Euphorbia decipiens, Phellodndron amurense, Salvia euphratica, Arabidopsis thaliana, Galinsoga quadriradiata, Cichorium pumilum*
SAK-T-27	Frangulin	*Rhamnus* spec.
SAK-T-28	Fraxin	*Aesculus hippocastanum, Fraxinus* spec., *Acer nikoense, Tetradium glabrifolium*
SAK-T-29	Gedunin	*Azadirachta indica, Cedrela odorata, Melia azedarach, Toona sinensis, Xylocarpus granatum, Entandrophragma angolense, Carapa guianesis*
SAK-T-30	Gentibiose	*Gentiana* spec., *Gentianopsis* spec., diverse stone fruits
SAK-T-31	Harmaline	*Peganum harmala, Banisteriopsis caapi, Passiflora* spec.
SAK-T-32	Harman	*Vestia foetida, Banisteriopsis caapi, Strychnos johnsonii, Ophiopogon* spec., *Carex brevicollis, Vitis vinifera*
SAK-T-33	Harmine	*Banisteriopsis caapi, Thalictrum foetidum, Peganum harmala, Passiflora incarnata, Festuca* spec.
SAK-T-34	Hesperetin	*Citrus* peels, *Brassica oleracea, Schizonepeta tenuifolia, Salvia* spec., *Arabidopsis thaliana, Rubus idaeus*
SAK-T-35	Hesperidin	*Citrus* peels, *Ficus erecta* var. *beecheyana, Zanthoxylum caribaeum, Betula pendula*
SAK-T-36	Heteropeucenin	*Cedrelopsis grevei*
SAK-T-37	Khellin	*Ammi visnaga, Annona muricata, Allium nutans, Dioscorea* spec.
SAK-T-38	Lawsone	*Lawsonia inermis, Eichhornia crassipes*
SAK-T-39	Lupeol	*Craveta nurvala, Betula* spec., *Ficus septica, Diospyros morrisiana, Paeonia emodi, Mikania haenkeana, Symphoricarpos albus, Avicennia officinalis, Derris trifoliata*
SAK-T-40	Morin	*Morus tinctoria, Lotus ucrainicus, Psidium guajava, Petasites formosanus, Tilia tomentosa, Maclura tricupidata, Plantago lanceolata, Endosamara racemosa*
SAK-T-41	Narceine	*Papaver somniferum*
SAK-T-42	Narcotine	*Papaver* spec.
SAK-T-43	Oleandrin	*Nerium oleander*
SAK-T-44	Ouabain	*Acocanthera schimperi, Acocanthera oppositifolia, Strophantus gratus, Cunila* spec.
SAK-T-45	Papaverine	*Papaver* spec., *Papaver rhoeas, Sauropus androgynus*
SAK-T-46	Peucenin-(5.7-dihydroxy-6-isopentyl-2-methylchromone	*Peucedanum ostruthium*
SAK-T-47	Physostigmine	*Datura stramonium, Physostigma venenosum, Hippomane mancinella*
SAK-T-48	Pilocarpine	*Pilocarpus* spec.,
SAK-T-49	Piperine	*Macropiper* spec., *Piper* spec.
SAK-T-50	Psoralen	*Ficu carica, Psoralea corylifolia, Citrus × limon, Angelica* spec., *Dianthus* spec., *Ammi visnaga, Pastinaca sativa, Petoselinum crispum, Levisticum officinale, Foeniculum vulgare, Daucus carota, Apium graveolens*
SAK-T-51	Ptaeroxylin	*Cedrelopsis gravei*
SAK-T-52	Quercetin (dihydrate)	Quercetin is ubiquitous in vegetarian food
SAK-T-53	Quinidine	Stereoisomer of quinine from *Cinchona officinalis*
SAK-T-54	Quinine (sulphate)	Quinine is from *Cinchona officinalis*
SAK-T-55	Rotenone	*Lonchocarpus nicou, Derris elliptica, Deguelia utilis*
SAK-T-58	Rutin	*Viola tricolor, Styphnolobium japonicum, Fagopyrum esculentum, Morus alba, Sambucus nigra* subsp. *canadensis, Petroselinum crispum, Persicaria hydropiper, Hypericum perforatum, Ficus virens, Visnea mocanera, Heliopsis helianthoides* var. *scabra, Ferulaga sylvatica, Nerium oleander, Polygonum cognatum*
SAK-T-59	Sanguinarine	*Sanguinaria canadensis, Argemone* spec., *Chelidonium majus, Macleaya cordata, Glaucium flavum, Fumaria* spec., *Eschscholzia california*
SAK-T-60	Santonin	*Artemisia* spec., *Fossombronia wondraczekii*
SAK-T-61	Scopoletin	*Scopolia* spec., *Artemisia* spec., *Viburnum prunifolium, Solanum nigrum, Urticaria dioica, Brunfelsia americana, Passiflora* spec., *Datura metel, Mallotus resinosus, Kleinhovia hospita, Trogonella foenum graecum, Taraxacum officinale, Ficus auriculata, Haplophyllum cappadocicum, Lessingianthus mollissimus, Zantoxylum ailanthoides, Tetradium glabrifolium, Phellodendron amurense*
SAK-T-62	Silibinin	*Silybum marianum, Anastatica hierochuntica, Silybum eburneum*
SAK-T-63	Sinigrin (monohydrate)	Sinigrin from *Cakile arabica, Erucaria cakiloidea, Brassica* spec., *Raphanus* spec., *Hirschfeldia incana*
SAK-T-64	Stigmasterol	*Physostigma venenosum, Glycine max, Brassica napus, Hebanthe eriantha, Xylopia aromatica, Citrus* spec., *Ophiopogon japonicus, Mirabilis jalapa*
SAK-T-65	Strophanthidin	*Strophantus* spec., *Convallaria majalis, Adonis* spec., *Apocrynum cannabinum, Descurainia sophia, Antiaris toxicaria, Cryptolepis migriscens, Erysimum inconspicuum*
SAK-T-66	Strychnine	*Strychnos nux-vomica, Ignatia amara*
SAK-T-67	Thebaine alkaloid	Thebaine from *Papaver bracteatum*
SAK-T-68	Ursolic acid	*Rosmarinus officinalis, Thymus vulgaris, Mirabilis jalapa, Malus domestica, Ocimum* spec., *Vaccinium* spec., *Sambucus* spec., *Crataegus* spec., *Lavandula spica, Origanum vulgare, Mentha × piperita, Prunus domestica, Pimpinella major, Gladiolus italicus, Symphoricarpus albus, Derri trifoliata, Rhizophora mucronata, Avicennia* spec., *Thymus* spec., *Nepeta cataria, Eucalyptus gradis, Psidium guajava, Prunella vulgaris, Nerium oleander, Ilex paraguariensis*
SAK-T-69	Vincamine	*Vinca* spec.
SAK-T-70	Yohimbine (hydrochloride)	*Corynanthe (Pausinystalia) johimbe*, *Rauvolfia* spec., *Aspidosperma quebracho-blanco, Tabernaemontana corymbosa, Alstonia angustifolia, Pouteria* spec.
SAK-T-71	β-Escin	*Aesculum hippocastanum, Bobgunnia madagascariensis*
SAK-T-72	P-Arbutin	*Viburnum opulus, Bergenia crassifolia, Schisandra chinensis, Grevillea robusta, Halocarpus biformis, Arabidopsis thaliana, Rhodolia* spec., *Vitis vinifera, Eriosema tuberosum*

**Table 2:** Acute and chronic toxicity of selected phytochemicals. The data were mined from the PubChem database (https://pubchem.ncbi.nlm.nih.gov/compound; accessed on 9 September 2022).

Phytochemical	Acute Toxicity *	Chronic Toxicity
Colchicine	Cardiotoxicity, hepatotoxicity, nephrotoxicity, gastrointestinal toxicity, lung toxicity, behavioral toxicity	None
Digitoxin	Cardiotoxicity, neurotoxicity, hematotoxicity, gastrointestinal toxicity, behavioral toxicity	None
Digoxin	Cardiotoxicity, hepatotoxicity, gastrointestinal toxicity, behavioral toxicity, lung toxicity	Carcinogenesis
Emetine	Cardiotoxicity, gastrointestinal toxicity, muscle weakness, dermal toxicity	Mutagenesis
Gedunin	Dermal toxicity (irritant)	None
Rotenone	Cardiotoxicity, gastrointestinal toxicity, lung toxicity, behavioral toxicity, dermal toxicity, neurotoxicity	None
Sanguinarine	Hepatotoxicity, behavioral toxicity	Teratogenesis, carcinogenesis
Strophantidine	Cardiotoxicity, lung toxicity, behavioral toxicity	Cardiotoxicity

* The information was extracted from PubChem.

**Table 3:** Molecular docking of gedunin to three different drug-binding sites of β-tubulin. Shown are the lowest binding energies (kcal/mol), predicted inhibition constants (µM), and amino acids involved in the ligand interaction of gedunin in comparison to the control drugs vinorelbine, paclitaxel, and colchicine, which are known to bind to the three binding sites.

Tubulin Binding Sites	Compounds	Lowest Binding Energy (Kcal/mol)	pKi (µM)	Amino Acids Involved in Ligand Interaction
*Vinca* alkaloid binding site	Vinorelbine	−10.76 ± 0.35	0.02 ± 0.01	THR210, PHE214, PRO222, TYR224.
	Gedunin	−8.89 ± 0.00	0.30 ± 0.00	GLN11, THR145, ASN206.
Taxane binding site	Paclitaxel	−10.41 ± 0.66	0.07 ± 0.04	LEU217, HIS229, PHE272, LEU275, THR276, GLN281, LEU371.
	Gedunin	−7.48 ± 0.00	3.27 ± 0.01	LEU217, LEU230, LEU275, LEU371, LYS372.
Colchicine binding site	Colchicine	−7.57 ± 0.04	2.82 ± 0.19	ASP69, ALA180, LEU248, LYS254.
	Gedunin	−4.49 ± 0.03	514.99 ± 2.92	ALA100, ASN101, THR179, GLU183, TYR224, LYS254.

**Table 4:** The separation of the clusters with cell lines derived from different tumor types is shown in Figure 6 according to their gedunin sensitivity or resistance using the χ^2^ test.

		Clusters 2,3,5		Clusters 1,4
Partition (log_10_IC_50_)		<−5.125 M		≥−5.125 M
Sensitive		17		6
Resistant		10		22
χ^2^ test: *p* = 0.002				
	Sensitive		Resistant	Suitability for therapy
Brain tumor	2		4	no
Renal Ca	3		5	no
Ovarian Ca	1		6	no
Melanoma	3		4	no
Breast Ca	4		1	yes
Lung Ca	3		6	no
Colon Ca	6		1	yes
Prostate Ca	2		0	yes
Leukemia	3		1	yes
χ^2^ test: *p* = 0.076				

**Table 5:** Correlation of the log_10_IC_50_ values for gedunin to ABC-transporter-mediated mechanisms of multidrug resistance (P-glycoprotein/ABCB1, ABCB5, ABCC1, and ABCG2) and other mechanisms of anticancer drug resistance (EGFR, RAS, TP53, WT1, HSP90, GSTπ, and the proliferative rate) in the NCI panel of tumor cell lines.

		Gedunine	Control Drug
		(log_10_ IC_50_, M)	(log_10_ IC_50_, M)
**ABCB1 Expression**			Epirubicin
7q21 (Chromosomal	*r*-value	−0.078	* **0.447**
locus of *ABCB1* gene)	*p*-value	0.297	* **3.55** **×** **10^−4^**
*ABCB1* expression	*r*-value	−0.097	* **0.533**
(microarray)	*p*-value	0.243	* **6.82** **×** **10^−6^**
*ABCB1* expression	*r*-value	−0.143	* **0.410**
(RT-PCR)	*p*-value	0.168	* **1.54** **×** **10^−3^**
Rhodamine 123	*r*-value	−0.115	* **0.526**
accumulation	*p*-value	0.207	* **1.12** **×** **10^−5^**
ABCB5 Expression			Maytansine
*ABCB5* expression	*r*-value	−0.072	* **0.454**
(microarray)	*p*-value	0.301	* **6.67** **×** **10^−4^**
*ABCB5* expression	*r*-value	0.036	* **0.402**
(RT-PCR)	*p*-value	0.396	* **0.0026**
ABCC1 Expression			Vinblastine
DNA gene	*r*-value	−0.072	* **0.429**
copy number	*p*-value	0.301	* **0.001**
*ABCC1* expression	*r*-value	0.051	* **0.399**
(microarray)	*p*-value	0.357	* **0.003**
*ABCC1* expression	*r*-value	0.061	**0.299**
(RT-PCR)	*p*-value	0.346	* **0.036**
ABCG2 Expression			Pancratistatin
*ABCG2* expression	*r*-value	−0.178	* **0.329**
(microarray)	*p*-value	0.099	* **0.006**
ABCG2 expression	*r*-value	−0.222	* **0.346**
(western blot)	*p*-value	0.052	* **0.004**
EGFR Expression			Erlotinib
*EGFR* gene	*r*-value	0.172	**−0.245**
Copy number	*p*-value	0.105	* **0.029**
*EGFR* expression	*r*-value	0.229	* **−0.458**
(microarray)	*p*-value	* 0.046	* **1.15** **×** **10^−4^**
*EGFR* expression	*r*-value	* 0.346	* **−0.379**
(PCR slot blot)	*p*-value	* 0.005	* **0.002**
EGFR expression	*r*-value	0.156	* **−0.376**
(protein array)	*p*-value	0.128	* **0.001**
TP53 Mutation			5-Fluorouracil
*TP53* mutation	*r*-value	−0.093	* **−0.502**
(cDNA sequencing)	*p*-value	0.253	* **3.50** **×** **10^−5^**
TP53 function	*r*-value	−0.115	* **−0.436**
(yeast functional assay)	*p*-value	0.213	* **5.49** **×** **10^−4^**
WT1 Expression			Ifosfamide
WT1 expression	*r*-value	−0.155	* **−0.316**
(microarray)	*p*-value	0.129	* **0.007**
GSTP1 Expression			Etoposide
*GSTP1* expression	*r*-value	0.009	**0.399**
(microarray)	*p*-value	0.473	* **9.58** **×** **10^−4^**
*GSTP1* expression	*r*-value	0.123	**0.509**
(northern blot)	*p*-value	0.185	* **2.24** **×** **10^−5^**
HSP90			Geldanamycin
*HSP90* Expression	*r*-value	* −0.426	* **−0.392**
(microarray)	*p*-value	* 5.92×10^−4^	* **0.001**
Proliferation			
Cell doubling	*r*-value	* 0.456	* **0.627**
	*p*-value	* 2.67 × 10^−4^	* **7–14** **×** **10^−6^**
*N-/K-/H-RAS* Mutations			Melphalan
*TP53* mutation	*r*-value	0.275	* **0.367**
(cDNA sequencing)	*p*-value	0.021	* **0002**

* *p* < 0.05 and *r* > 0.3 (or *r* < −0.3).

## Data Availability

Not applicable.

## Supplementary Materials

The following are available online at https://www.mdpi.com/article/10.3390/molecules27185858/s1. Figure S1: Cytotoxicity of Gedunin on PBMCs. Each point illustrates the mean value ± SD of two independent experiments with six replicates each. Table S1: Characteristics of phytochemicals with SAK-T code. Table S2: Pearson correlation test-based COMPARE analysis of proteins directly or inversely correlating with the log10IC50 values of gedunin in 55 tumor cell lines.
