# Supplementary material for: Identification of Gedunin from a Phytochemical Depository as a Novel Multidrug Resistance-Bypassing Tubulin Inhibitor of Cancer Cells
Source: Molecules. 2022 Sep 9;27(18):5858. doi: 10.3390/molecules27185858 (PMC9501561; doi:10.3390/molecules27185858)
Supplement: Supplementary file 1 [file molecules-27-05858-s001.zip › molecules-1867972-supplementary.pdf]

Supplementary file

Identification of gedunin from a phytochemical depository as a novel multidrug resistance-bypassing tubulin inhibitor of cancer cells

Sami A. Khalid[1,*], Mona Dawood[2,4], Joelle C. Boulos[2], Monica Wasfi[1], Assia Drif[2], Mohamed E. M. Saeed[2], Faranak Bahramimehr[2], Nasim Shahhamzehei[2], Letian Shan[3], Thomas Efferth[2,*]

[1] Faculty of Pharmacy, University of Science & Technology, Omdurman, Sudan
[2] Department of Pharmaceutical Biology, Institute of Pharmaceutical and Biomedical Sciences, Johannes Gutenberg University, Staudinger Weg 5, 55128 Mainz, Germany
[3] The First Affiliated Hospital, Zhejiang Chinese Medical University, Hangzhou, 310053, Zhejiang, China
[4] Home address: Department of Molecular Biology, Faculty of Medical Laboratory Sciences, Al-Neelain University, Khartoum, Sudan

* Correspondence:
S. Khalid (E-mail: khalidseek@hotmail.com)
T. Efferth (E-mail: efferth@uni-mainz.de)

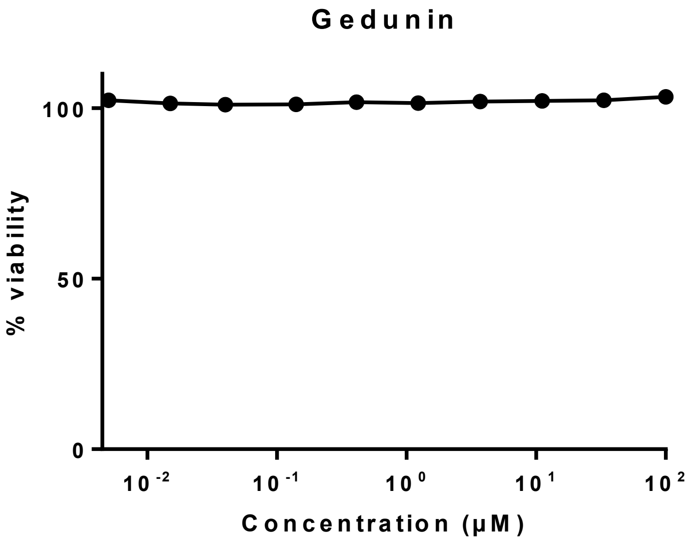

Supplementary Figure S1: Cytotoxicity of Gedunin on PBMCs. Each point illustrates the mean value ± SD of two independent experiments with six replicates each.

Supplementary Table SI: Characteristics of phytochemicals with SAK-T code.

Code	Compound	MW	CID Code	2D Structure
SAK-T1	(-)-Scopolamine hydrochloride	339.82	6852406	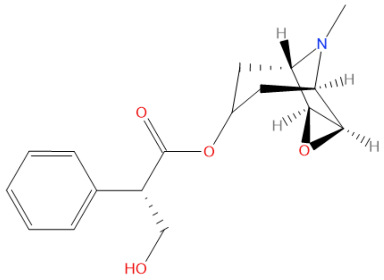
SAK-T2	(+)-Bicuculline	367.35	10237	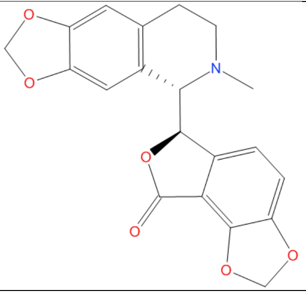
SAK-T3	(+)-Laudanosine	357.45	73397	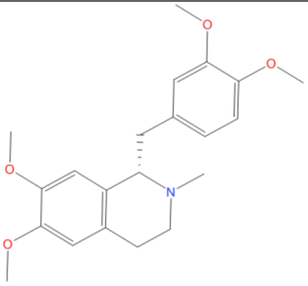
SAK-T4	(+)-Tubocurarine	609.74	6000	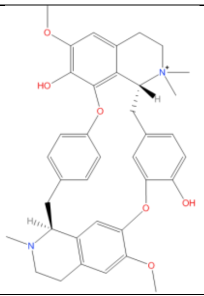
SAK-T5	(+)-Usnic acid	344.31	442614	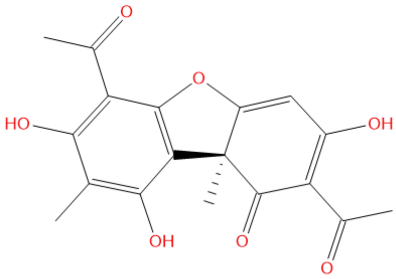
SAK-T6	1,8-Dihydroxyanthraquinone	240.21	2950	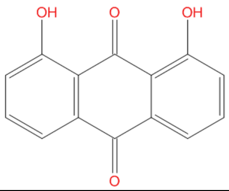

SAK-T7	18-beta-Glycyrrhetinic acid	470.69	10114	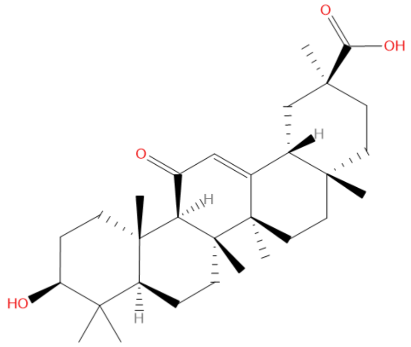
SAK-T8	3,5-Dimethoxy-4-hydroxycinnamic acid	224.21	637775	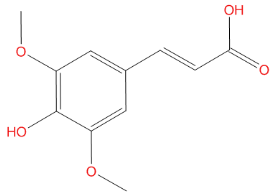
SAK-T9	Alloptaeroxylin	258.27	12305984	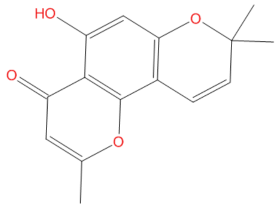
SAK-T10	Apigenin	270.24	5280443	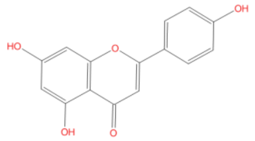
SAK-T11	Aristolochic acid	341.27	2236	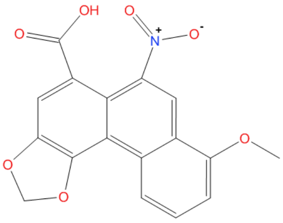
SAK-T12	Barbaloin	418.398	12305761	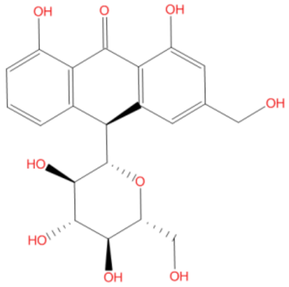
SAK-T13	Berberine	371.81	2353	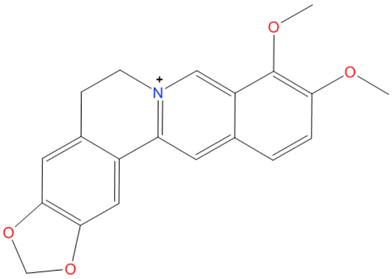

SAK-T14	Boldine	327.38	10154	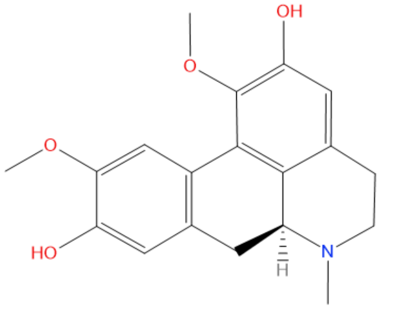
SAK-T15	Colchicine	399.44	6167	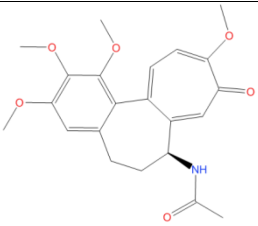
SAK-T16	Cryptopine	369.41	72616	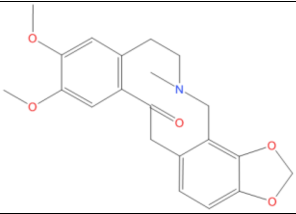
SAK-T17	Cumarin	146.14	323	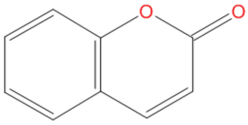
SAK-T18	Digitoxin	764.95	441207	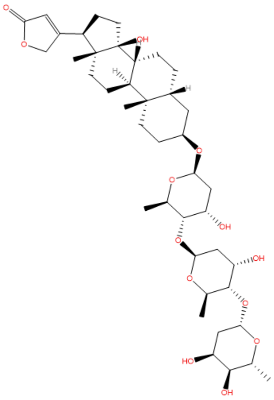
SAK-T19	Digoxin	780.94	2724385	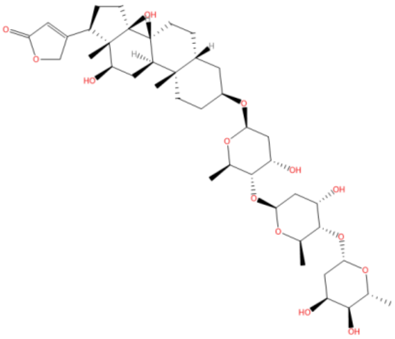

SAK-T20	Diosgenin	414.63	99474	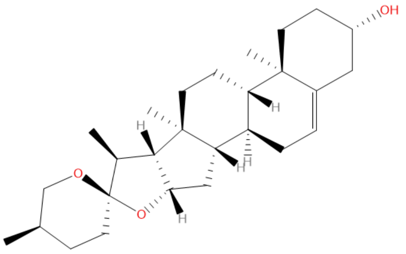
SAK-T21	Emetine dihydroch loride	553.56	3068143	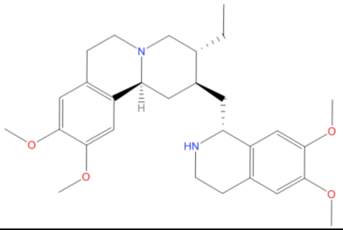
SAK-T22	Emodin	270.24	3220	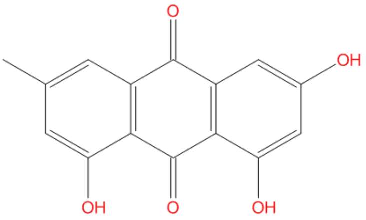
SAK-T23	Emodin anthrone	256.26	122635	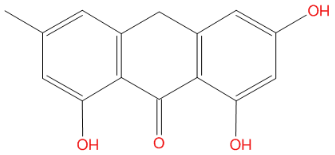
SAK-T25	Esculetin	178.14	5281416	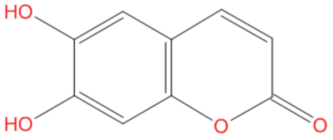
SAK-T26	Esculin	340.28	5281417	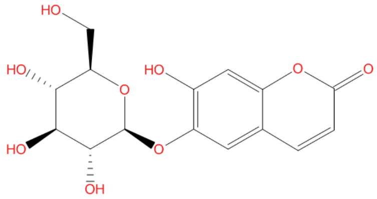
SAK-T27	Frangulin	416.37	348160	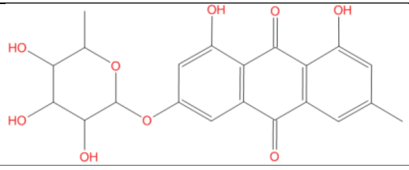
SAK-T28	Fraxin	370.31	5273568	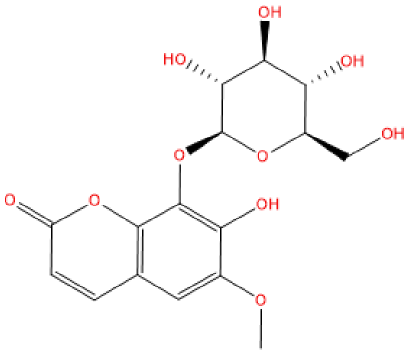

SAK-T29	Gedunine	482.57	12004512	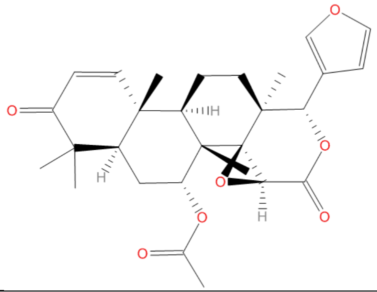
SAK-T30	Gentibiose	342.30	5460026	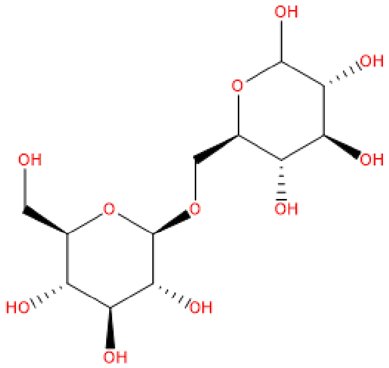
SAK-T31	Harmaline	214.26	3564	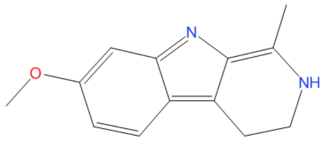
SAK-T32	Harman	182.22	5281404	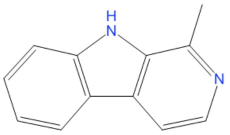
SAK-T33	Harmine	212.25	5280953	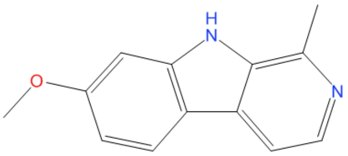
SAK-T34	Hesperetin	302.282	72281	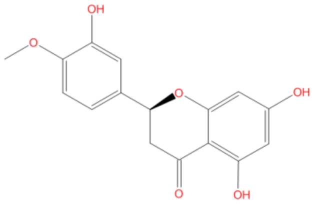
SAK-T35	Hesperidin	610.57	10621	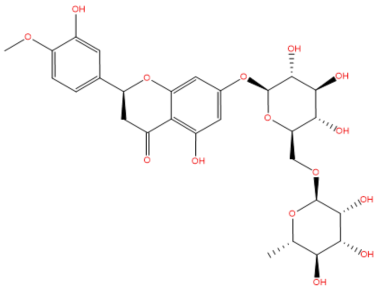

SAK-T36	Heteropeucenin	260.29	12310443	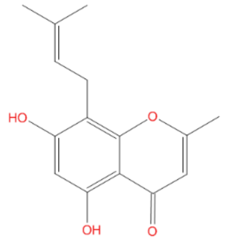
SAK-T37	Khellin	260.24	3828	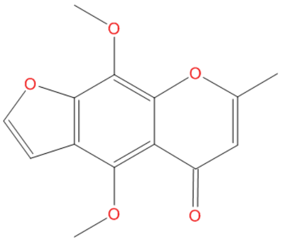
SAK-T38	Lawsone	174.15	6755	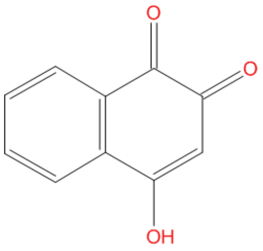
SAK-T39	Lupeol	426.72	259846	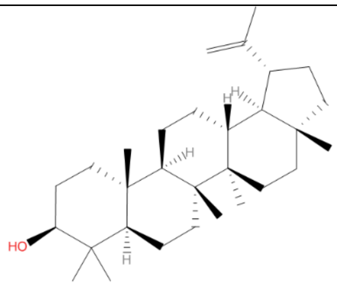
SAK-T40	Morin	302.24	5281670	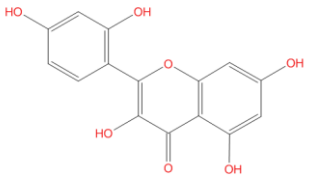
SAK-T41	Narceine	445.47	8564	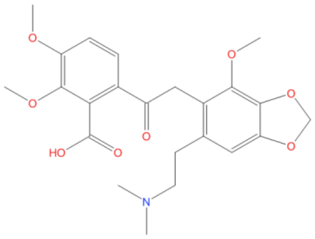
SAK-T42	Narcotine	413.42	4544	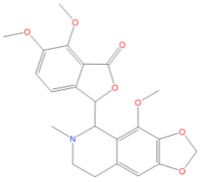

SAK-T43	Oleandrin	576.72	11541511	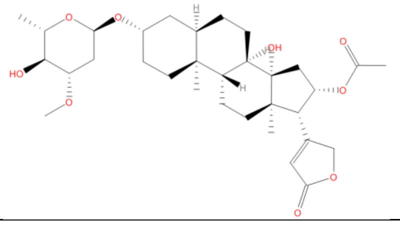
SAK-T45	Papaverine	375.85	6084	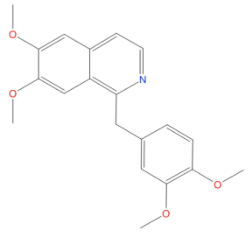
SAK-T46	Peucenin, (5,7-dihydroxy-6-isopentyl-2-methylchromone)	274.12	68477	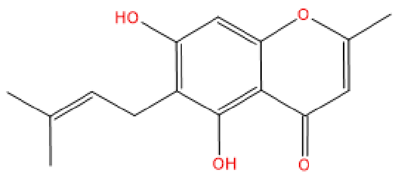
SAK-T47	Physostigmine	275.35	5983	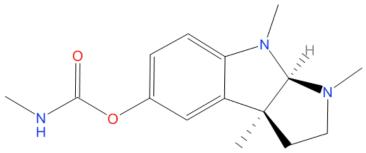
SAK-T48	Pilocarpine	208.26	5910	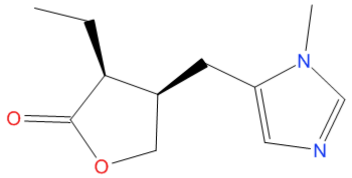
SAK-T49	Piperine	285.34	638024	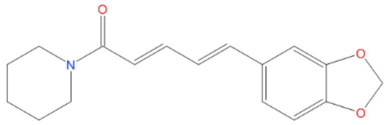
SAK-T50	Psoralen	186.16	6199	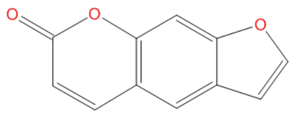
SAK-T51	Ptaeroxylin	258.27	3646533	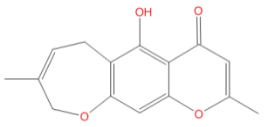

SAK-T52	Quercetin dihydrate	338.27	5280343	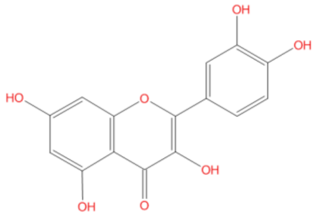
SAK-T53	Quinidine	324.42	441074	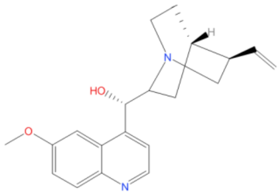
SAK-T54	Quinine sulphate	782.96	16211610	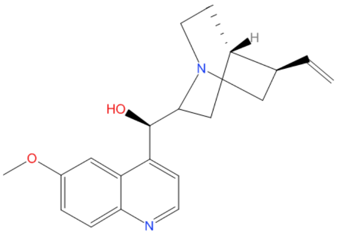
SAK-T55	Rotenone	394.42	6758	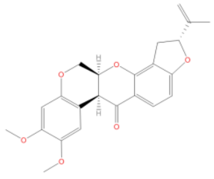
SAK-T58	Rutin	610.52	5280805	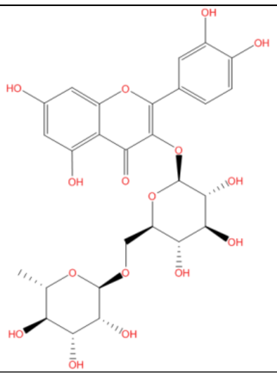
SAK-T59	Sanguinarine	367.78	68635	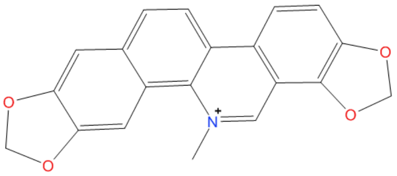
SAK-T60	Santonin	246.30	221071	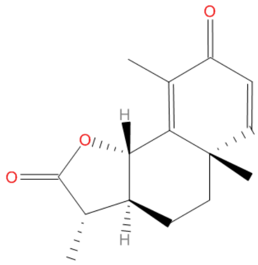

SAK-T61	Scopoletine	192.17	5280460	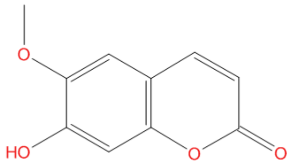
SAK-T62	Silibinin	482.441	31553	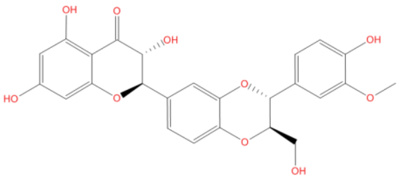
SAK-T63	Sinigrin monohydrate	415.47	23670774	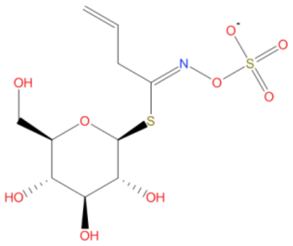
SAK-T64	Stigmasterol	412.69	5280794	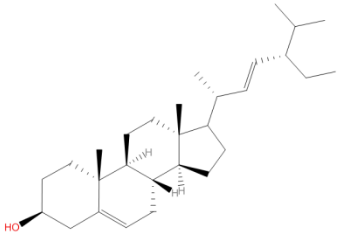
SAK-T65	Strophanthidin	404.5	6185	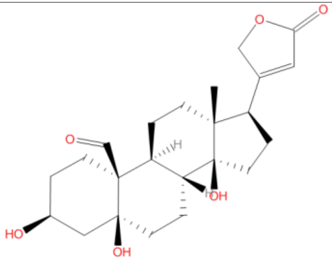
SAK-T66	Strychnine	334.42	441071	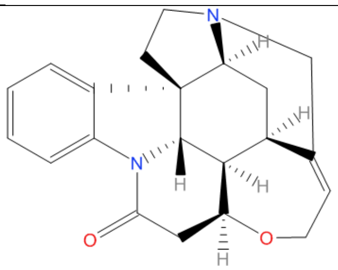
SAK-T67	Thebaine alkaloid	311.38	5321926	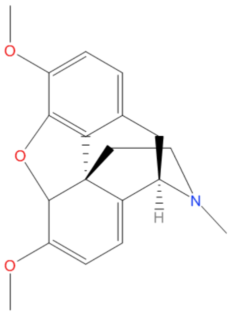

SAK-T68	Ursolic acid	456.71	64945	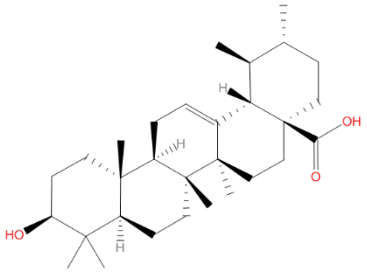
SAK-T69	Vincamine	354.44	15376	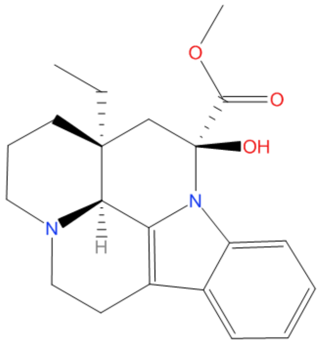
SAK-T70	Yohimbine hydrochloride	390.91	6169	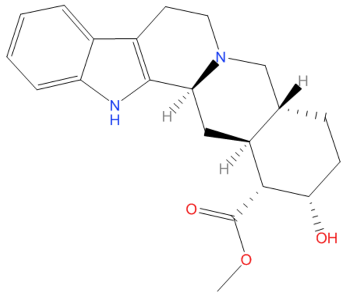
SAK-T71	*β*-Escin	1131.26	6540709	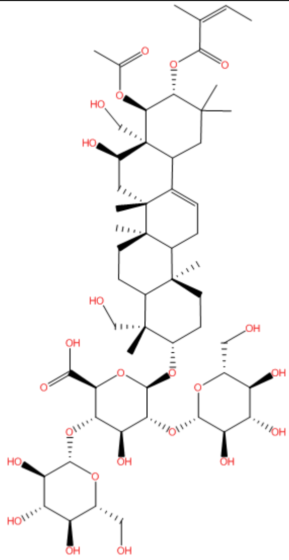

SAK-T72	P-Arbutin	272.25	440936	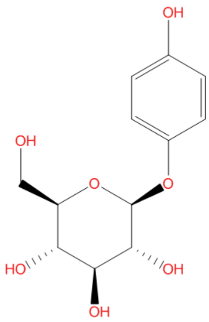

Supplementary Table S2. Pearson correlation test-based COMPARE analysis of proteins directly or inversely correlating with the $log_{10}IC_{50}$ values of gedunin in 55 tumor cell lines. The protein functions have been extracted from the GeneCards database (https://www.genecards.org).

Symbol	*r*-Value	*p*-Value	Entry Gene ID	Name	Function
**MOGS**	0.474	1.28×10^{-4}	7841	Mannosyl-oligosaccharide glucosidase	Cleavage of glucose from oligosaccharide precursor
**PDS5A**	0.454	2.51×10^{-4}	23244	PDS5 cohesin-associated factor A	Regulation of sister chromatid cohesion during mitosis
**IKBKAP**	0.438	4.15×10^{-4}	8518	Elongator acetyltransferase complex subunit 1	Binding of NF-κB-inducing kinase and I-κB kinases to active the kinase complex
**SLC12A4**	0.432	4.90×10^{-4}	6560	Solute carrier family 12 member 4	Coupled movement of potassium and chloride ions across the plasma membrane
**SPECC1**	0.425	6.17×10^{-4}	92521	Sperm antigen with calponin homology and coiled-coil domains 1	May cause juvenile myelomonocytic leukemia.
**GDF15**	0.419	7.22×10^{-4}	9518	Growth differentiation factor 15	Binding of TGF-β receptors leads to the recruitment and activation of SMAD family transcription factors for gene expression. Involved in the stress response program of cells after cellular injury
**NUDC**	0.402	1.16×10^{-3}	10726	Nuclear distribution C, dynein complex regulator	Spindle formation in mitosis and microtubule organization during cytokinesis.
**PA2G4**	0.397	1.32×10^{-3}	5036	Proliferation-associated 2G4	Ribosome assembly and regulation of rRNA processing. Transcriptional co-repressor of cell cycle genes by interacting with histone deacetylases. Implicated in growth inhibition and differentiation of cancer cells
**IMPDH2**	0.386	1.78×10^{-3}	3615	Inosine monophosphate dehydrogenase 2	De novo guanine nucleotide biosynthesis for DNA and RNA synthesis. Role in malignant transformation
**RPS28**	0.384	1.88×10^{-3}	6234	Ribosomal protein S28	Involved in ribosomal protein synthesis
**PEA15**	0.377	2.27×10^{-3}	8682	Proliferation and apoptosis adaptor protein 15	Negative apoptosis regulator.
**REEP5**	0.376	2.35×10^{-3}	7905	Receptor accessory protein 5	Involved in endoplasmic reticulum organization and regulation of intracellular transport
**HM13**	0.374	2.43×10^{-3}	81502	Histocompatibility minor 13	Intramembrane proteolysis of signal peptides
**PGRMC1**	0.366	2.96×10^{-3}	10857	Progesterone receptor membrane component 1	Putative membrane-associated progesterone steroid receptor

**B2M**	0.365	3.08×10^{-3}	567	β-2-Microglobulin	Serum protein associated with the major histocompatibility complex (MHC) class I heavy chain
**AQR**	0.365	3.09×10^{-3}	9716	Aquarius intron-binding spliceosomal factor	Involved in mRNA splicing via spliceosome
**HNRNPC**	0.364	3.17×10^{-3}	3183	Heterogeneous nuclear ribonucleoprotein C	RNA-binding protein influencing pre-mRNA processing
**PSMA7**	0.359	3.55×10^{-3}	5688	Proteasome 20S subunit α7	Part of the 26S proteasome. Role in the cellular stress response by regulating hypoxia-inducible factor-1α
**EIF3G**	0.359	3.57×10^{-3}	8666	Eukaryotic translation initiation factor 3 subunit G	Initiation of protein translation
**TRAP1**	0.358	3.67×10^{-3}	10131	Tumor necrosis factor type 1 receptor-associated protein 1	Mitochondrial chaperone and member of the heat shock protein 90 (HSP90) family. Regulator of cellular stress responses
**DDX21**	-0.483	9.22×10^{-5}	9188	DEAD (Asp-Glu-Ala-Asp) box helicase 21	Putative RNA helicase implicated in alterations of the RNA secondary structure. Involved in cellular growth and division
**MSH2**	-0.475	1.25×10^{-4}	4436	MutS (*E. coli*) homolog 2 (colon cancer, nonpolyposis type 1)	Mismatch repair gene mutS
**UBE2S**	-0.469	1.54×10^{-4}	27338	Ubiquitin conjugating enzyme E2 S	Ubiquitin-conjugating enzyme and ubiquitin carrier protein
**DRG1**	-0.460	2.05×10^{-4}	4733	Developmentally regulated GTP binding protein 1	Positive regulation of microtubule polymerization and mitotic spindle assembly
**EIF3I**	-0.457	2.25×10^{-4}	8668	Eukaryotic translation initiation factor 3 subunit I	Involved in translational initiation
**NEBL**	-0.453	2.61×10^{-4}	10529	Nebulette	Binding of actin and interaction with thin filaments and Z-line-associated proteins in striated muscle
**EIF3E**	-0.454	2.52×10^{-4}	3646	Eukaryotic translation initiation factor 3 subunit E	Positive regulation of mRNA binding activity, gene expression, and translational initiation
**CARM1**	-0.453	2.54×10^{-4}	10498	Coactivator-associated arginine methyltransferase 1	Methylation of histones and other chromatin-associated proteins. Regulation of gene expression
**PPP2R1B**	-0.452	2.66×10^{-4}	5519	Protein phosphatase 2 (formerly 2A), regulatory subunit A (PR 65), β isoform	Ser/Thr phosphatase implicated in the negative control of cell growth and division
**AP2A1**	-0.450	2.80×10^{-4}	160	Adaptor-related protein complex 2 subunit α1	Component of the adaptor protein complex 2 (AP-2). Protein transport via transport vesicles in different membrane traffic pathways

**SEC22B**	-0.448	2.97×10^{-4}	9554	SEC22 vesicle trafficking protein homolog B (*S. cerevisiae*)	Endoplasmic reticulum to Golgi protein trafficking
**YIPF5**	-0.447	3.15×10^{-4}	81555	Yip1 domain family member 5	Regulation of endoplasmic reticulum to Golgi vesicle-mediated transport
**ITGAV**	-0.446	3.22×10^{-4}	3685	Integrin, Alpha V (vitronectin receptor, α polypeptide, antigen CD51)	Cell surface adhesion and signaling. Regulation of angiogenesis and cancer progression
**ABCD3**	-0.445	3.30×10^{-4}	5825	ATP-binding cassette subfamily D member 3	Role in peroxisome biogenesis by fatty acid transport
**GNAI3**	-0.445	3.31×10^{-4}	2773	Guanine nucleotide-binding protein G(I) subunit α-3	Transmembrane signal transduction.
**MBOAT7**	-0.443	3.51×10^{-4}	79143	Membrane-bound O-acyltransferase domain-containing 7	Membrane-bound O-acyltransferase domain-containing 7
**GNAI1**	-0.440	3.86×10^{-4}	2770	Guanine nucleotide-binding protein G(I) subunit α1	Signal transduction of β-adrenergic signals by inhibiting adenylate cyclase
**MMGT1**	-0.438	4.11×10^{-4}	93380	Membrane magnesium transporter 1	Membrane insertase activity for endoplasmic reticulum proteins
**EIF3H**	-0.438	4.16×10^{-4}	8667	Eukaryotic translation initiation factor 3 subunit H	Deubiquitinase and translation initiation factor activities. Implicated in breast, prostate, and prostate carcinoma
**FAF2**	-0.436	4.38×10^{-4}	91942	Fas-associated factor family member 2	Apoptosis resistance to apoptosis in T cells and eosinophils
